# High performance p-type organic thin film transistors with an intrinsically photopatternable, ultrathin polymer dielectric layer^☆^

**DOI:** 10.1016/j.orgel.2013.07.014

**Published:** 2013-11

**Authors:** Andreas Petritz, Archim Wolfberger, Alexander Fian, Joachim R. Krenn, Thomas Griesser, Barbara Stadlober

**Affiliations:** a Materials-Institute for Surface Technologies and Photonics, Franz-Pichler Straße 30, Weiz A-8160, Austria; b Chemistry of Polymeric Materials, University of Leoben, Otto Glöckel-Straße 2, Leoben A-8700, Austria; c Institute of Physics, Universitätsplatz 5, Graz A-8010, Austria

**Keywords:** Dielectrics, Thin films, Organic field effect transistor, Organic electronics, Photochemistry

## Abstract

A high-performing bottom-gate top-contact pentacene-based oTFT technology with an ultrathin (25–48 nm) and electrically dense photopatternable polymeric gate dielectric layer is reported. The photosensitive polymer poly((±)endo,exo-bicyclo[2.2.1]hept-5-ene-2,3-dicarboxylic acid, diphenylester) (PNDPE) is patterned directly by UV-exposure (*λ *= 254 nm) at a dose typical for conventionally used negative photoresists without the need for any additional photoinitiator. The polymer itself undergoes a photo-Fries rearrangement reaction under UV illumination, which is accompanied by a selective cross-linking of the macromolecules, leading to a change in solubility in organic solvents. This crosslinking reaction and the negative photoresist behavior are investigated by means of sol–gel analysis. The resulting transistors show a field-effect mobility up to 0.8 cm^2^ V^−1^ s^−1^ at an operation voltage as low as −4.5 V. The ultra-low subthreshold swing in the order of 0.1 V dec^−1^ as well as the completely hysteresis-free transistor characteristics are indicating a very low interface trap density. It can be shown that the device performance is completely stable upon UV-irradiation and development according to a very robust chemical rearrangement. The excellent interface properties, the high stability and the small thickness make the PNDPE gate dielectric a promising candidate for fast organic electronic circuits.

## Graphical abstract

**Graphical abstract:**

**Figure:**
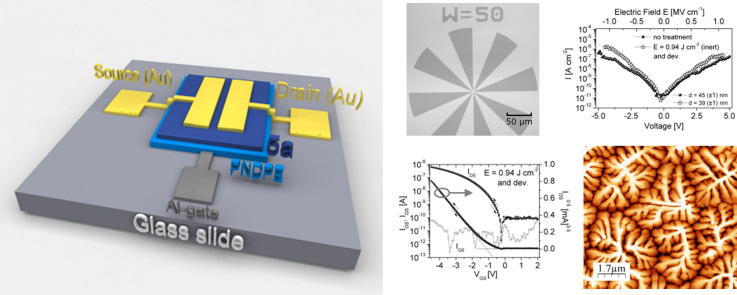

**Highlights:**

• Low operation voltage oTFT with ultrathin photopatternable polymer gate dielectric.
• Intrinsically UV-crosslinkable without the usage of any photoinitiator.
• Low leakage current in the range of 10^−6^ A cm^−2^ at 1 MV cm^−1^.
• Very low interface trap density in the range of 5 × 10^11^ cm^−2^ eV^−1^.
• Transistor performance is stable upon UV-irradiation and wet chemical development.

## 1 Introduction

The direct integration of disposable organic electronics could make everyday things “smarter”, thus opening a wide field of novel applications such as flexible RFID tags [1], electronic paper [2], intelligent textiles [3] or bioelectronics [4]. In the context of ambient intelligence, the simplicity of processing is one of the key issues for true low-cost fabrication of organic circuits [5]. Only then the compatibility with a variety of substrate materials like plastics foils, textiles or even paper is ensured. Usage of an intrinsic photopatternable gate dielectric in organic thin film transistors (oTFT) – one of the basic elements for any organic logic circuit-clearly simplifies the fabrication of via holes and therefore the design of circuits. However, the electronic performance of the oTFTs has to fulfill the requirements of practical life. One important issue here is the achievement of a low oTFT supply voltage so that the organic electronic circuit can be powered by household batteries [6].

**Fig. 1:**
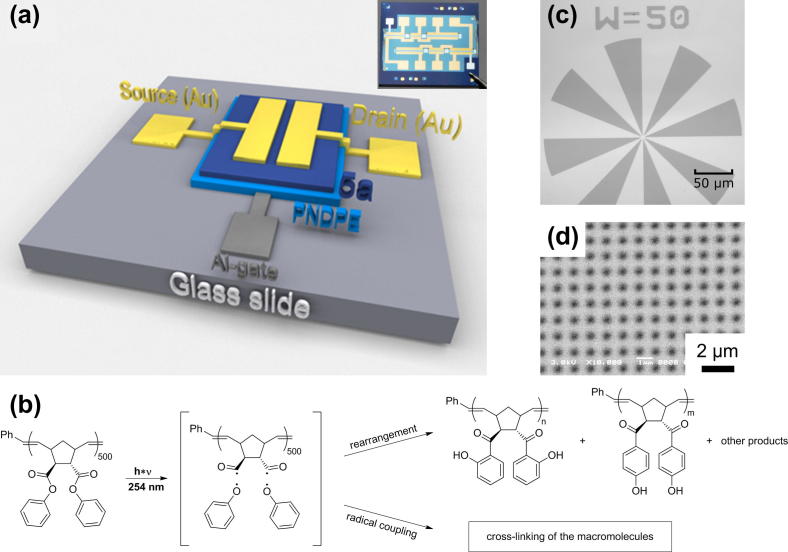
(a) Schematic structure of the fabricated oTFTs, showing the glass substrate (light grey), aluminium gate electrode (dark grey), PNDPE dielectric (light blue), pentacene layer (dark blue) and Au source- and drain electrodes (yellow). The inset in Fig. 1a shows 6 oTFT’s with a PNDPE layer photopatterned through a shadow mask in order to open the gate contact pad. A SiO_2_ wafer is used here as a substrate for a clearer illustration of the patterned dielectric layer. (b) Schematic mechanism of photochemical reactions upon UV irradiation within PNPDE. Upon irradiation with UV light, photolysis leads to the cleavage of the aromatic ester in the polymer side chain, resulting in the formation of polymeric acyl radicals and phenoxy radicals. Recombination of these two radicals leads to the corresponding photo-Fries product, i.e. ortho-hydroxyketone (photo Fries rearrangement), whereas the radical coupling reaction of two polymeric acyl radicals results in crosslinking. (c) A micrograph of a photopatterned PNDPE polymer. The pattering is done under ambient condition and an exposure dose of 1.56 J cm^−2^ is used. (d) A scanning electron microscopy (SEM) image of a PNDPE dot array pattern structured by means of e-beam lithography. (For interpretation of the references to colour in this figure legend, the reader is referred to the web version of this article.)

The charge separation and, accordingly, the charge carrier density in the source–drain channel of an oTFT (Fig. 1a) are induced by the vertical electric field that is established between the gate-electrode and the semiconductor layer and drops over the gate dielectric. High charge carrier densities at low gate voltage and thus full transistor operation can be achieved only for high capacitive dielectrics with low leakage currents. In previous works, different approaches for high capacitive gate dielectrics were reported: one is the usage of ultra-thin close packed organic layers with a thickness of only a few nanometers, built up by self-assembling monolayers (SAMs) [6] or polyelectrolytes [7].

In a second approach high-k-dielectrics like titanium oxide (TiO_2_), hafnia (HfO_2_), tantalum oxide (Ta_2_O_3_), alumina (Al_2_O_3_) or zirconia (ZrO_2_) fabricated by atomic layer deposition [8] and in a third approach also oxide/polymer bilayers and nanocomposites were utilized [9]. The applicability of ultrathin high-k polymer dielectric layers for low voltage organic thin film transistors (but without photostructuring) was shown in [10], [11].

Since these approaches require complex photopatterning processes of the gate dielectric layer, which is essential to establish the electrical interconnection in electronic circuits, photoresists such as SU-8 were proposed as an alternative [12]. However, polymeric dielectric materials which contain photo-polymerizable units such as epoxy groups in SU-8 require photoinitiators for the light-induced curing. These photosensitive molecules lead to the formation of either reactive radicals or acidic groups initiating the polymerization and thus the crosslinking upon UV irradiation. In particular, residual ionic photoinitiators such as Crivello salts and also their cleavage products in the gate dielectric may cause hysteresis effects. It is well known that mobile ionic impurities can be responsible for the hysteresis in oTFTs and also decrease the long term stability of such devices [13]. Very often also an immediate deterioration of the oTFT characteristics was observed in photoinitiator containing polymer dielectrics that was caused by the UV-illumination step [14]. For the remaining reported photoinitiator-free UV-patternable dielectrics complex multistep cross-linking and curing processes of polymer blends had to be used to achieve thin and electrically dense gate dielectrics [15].

In this work, we explore the use of poly((±)endo,exo-bicyclo[2.2.1]hept-5-ene-2,3-dicarboxylic acid, diphenylester) (PNDPE) as a directly photopatternable dielectric material for low voltage pentacene based oTFTs. This polymer bears aromatic ester groups which are capable of undergoing the photo-Fries rearrangement upon irradiation with UV-light.

The photo-Fries rearrangement, as first observed by Anderson and Reese [16] leads to a photolytic cleavage of the aryl ester moieties to acyl- and phenoxy radicals. The generated radicals can then recombine and form the corresponding ortho- and para-hydroxyketones. A schematic mechanism of the photo-Fries rearrangement reaction of PNDPE is depicted in Fig. 1b. In our previous work, we have studied this photoreaction in thin films of PNDPE by means of UV/VIS- and FTIR-spectroscopy extensively. In this context, also the influence of the formed photoproducts, i.e. hydroxyketones, on the refractive index and the surface properties was explored in detail [17], [18]. Although PNDPE layers have already been tested as gate dielectric material in fullerene based n-channel oTFTs, a strong degradation of the device characteristics was observed when fullerene were deposited on illuminated PNDPE layers [14]. However, compared to the present contribution this group used commensurably thick polymer layers of about 3 μm in thickness and the halogenated solvent chlorobenzene. Additionally, no devices with a photopatterned dielectric were demonstrated.

In our new approach the photopatterning process was realized without any additional photoinitiator by exploiting a known side reaction of this photoreaction of this polymeric material as illustrated in Fig. 1b [19]. The photogenerated acyl radicals can undergo a recombination reaction leading to a selective crosslinking of macromolecules. The polymer itself was dissolved in the low vapor pressure and non-halogenated solvent anisole. With this setup very homogenous high capacitive thin films with a thickness in the order of 25–48 nm could be achieved.

These high qualitative ultrathin PNDPE films were used as a dielectric layer in the fabrication of low-voltage pentacene based oTFTs. The influence of the photopatterning process under ambient and inert conditions on the dielectric and the surface properties as well as on the device characteristics was investigated in detail. Negative charges are generated by the photoinduced rearrangement reaction both under ambient and inert conditions inducing a positive threshold voltage shift, but the interface trap density remains very low. It turned out that the changes in surface energy and roughness, which are induced by a long-term UV-illumination under inert conditions are negligible and do not influence the semiconductor morphology or the mobility.

In devices with completely cross-linked dielectric layer (under inert conditions) stable hysteresis-free characteristics are observed with reasonably high mobilities up to 0.35 cm^2^ V^−1^ s^−1^ and small threshold voltages around −1 V.

PNDPE-layers which were UV-illuminated in ambient conditions show a significant change in surface energy and roughness. This leads to a change in semiconductor growth and mobility and can be attributed to the impact of UV-ozone on the unprotected polymer layer in our experimental setup. Nevertheless, oTFTs with a mobility up to 0.8 cm^2^ V^−1^ s^−1^ (but less reproducible) could be shown under these conditions.

## 2 Experimental

### 2.1 Cross linking behavior of PNDPE

The detailed synthesis of the photopatternable polymer is reported in Ref. [17]. Structured PNDPE films were fabricated by UV-irradiation in a conventional mask aligner system with the opportunity of nitrogen purge (SÜSS MJB4, 500W HgXe lamp) with a power density of 21.3 mW cm^−2^ by using a quartz chromium mask with a resolution test structure in hard contact mode (Fig. 1c). FT-IR spectra (photoinduced conversion, sol/gel fraction analysis) were performed with a Perkin Elmer “Spectrum One” instrument (spectral range between 4000 and 450 cm^−1^, resolution 1 cm^−1^). All FT-IR spectra were recorded in transmission mode.

### 2.2 Test samples for determination of the capacitance, the surface energy and pentacene growth

The thin PNDPE test layers were formed on pre-cleaned object slides with a 30 nm thick aluminum layer, e-beam evaporated under high vacuum conditions (UNIVEX 350 from Oerlikon), by spin-coating a solution of PNDPE in anisole with a concentration of 20 mg ml^−1^ at 3000 rpm for 30 s. Afterwards, a prebake at 60 °C for 90 min in vacuum serve to remove solvent residuals. The PNDPE layer was then UV-illuminated with an exposure dose of 0.39 J cm^−2^ (5 min UV-illumination), 0.94 J cm^−2^ (12 min UV-illumination), 1.56 J cm^−2^ (20 min UV-illumination), 2.34 J cm^−2^ (30 min UV-illumination) and 4.68 J cm^−2^ (60 min illumination) with a polychromatic Hg lamp (Benda NU 72). The UV-exposure was done under inert gas but also under ambient conditions in order to study the influence of photooxidation reactions. The light intensity (power density) of 1.3 mW cm^−2^ at the sample surface was measured with an optical power meter (Newport 842 PE) at a measuring range from 200 nm to 1100 nm. The development step of the illuminated samples (see below) was simulated by rinsing the samples in anisole for a few seconds. The layer thickness of the dielectric layers was determined by profilometer measurements (VEECO Dektak 150).

Contact angle measurements, conducted on a KRÜSS DSA 100 Contact Angle Measuring System, were used to determine the influence of the pattering process (UV-illumination and development) on the surface energy. The used liquids were ultrapure water (a very polar liquid with a surface tension *γ *= 72.8 mN m^−1^ separable in a polar component *γ^P^* = 51 mN m^−1^ and a dispersive component *γ^D^ *= 21.8 mN m^−1^) and diiodomethane (a very nonpolar liquid with *γ *= 50.8 mN m^−1^, *γ^P^ *= 0 mN m^−1^ and *γ^D^ *= 50.8 mN m^−1^). The surface energy is calculated from five droplets of water and diiodomethane, respectively, for each treatment of the PNDPE layer and the mean values from 5 samples with their standard derivations are compiled in Table 1.

**Table 1:** Calculated values of maximum interfacial trap density for differently treated PNDPE gate dielectric layers and calculated surface energy of treated and untreated PNDPE layers.

Sample^a^	*E* (J cm^−2^)^b^	Dev.	*D* (nm)^c^	*C_i_* (nF cm^−2^)^d^	*RMS* (nm)^e^	*S* (mV dec^−1^)^f^	*N_ss,max_* (cm^2^ eV^−1^)^g^	*γ* (mJ m^−2^)^h^	*γ^D^* (mJ m^−2^)^i^	*γ^P^* (mJ m^−2^)^i^	Surface Polarity (%)^j^
*Inert*											
A	–	–	42 (±1)	50	0.24 (±0.02)	110 (±20)	2.7 × 1011	44.5 (±1.7)	42.6 (±1.5)	1.9 (±0.2)	4.3 (±0.4)
B	0.39	–	43 (±1)	49	0.25 (±0.02)	140 (±20)	4.3 × 1011	44.3 (±0.5)	42.1 (±0.3)	2.2 (±0.2)	5.0 (±0.5)
C	0.94	–	43 (±1)	50	0.24 (±0.02)	150 (±20)	4.9 × 1011	43.7 (±1.4)	41.3 (±1.1)	2.4 (±0.3)	5.5 (±0.7)
D	1.56	–	43 (±1)	50	0.27 (±0.02)	220 (±20)	8.6 × 1011	44.1 (±0.9)	41.5 (± 0.6)	2.6 (± 0.3)	5.9 (±0.7)
E	2.34	–	42 (±1)	53	0.25 (±0.02)	195 (±20)	7.7 × 1011	45.0 (±1.0)	42.3 (±0.8)	2.7 (±0.2)	6.0 (±0.4)

F	4.68	–	42 (±1)	54	0.28 (±0.02)	140 (±20)	4.7 × 1011	45.3 (±1.9)	42.7 (±1.7)	2.6 (±0.2)	5.7 (±0.4)
G	0.39	Yes	33 (±1)	65	0.28 (±0.02)	100 (±20)	2.9 × 1011	–	–	–	–
H	0.94	Yes	35 (±1)	60	0.26 (±0.02)	100 (±20)	2.7 × 1011	44.9 (±0.75)	42.9 (±0.6)	2.0 (±0.15)	4.5 (±0.3)
I	1.56	Yes	39 (±1)	54	0.29 (±0.02)	110 (±20)	3.0 × 1011	45.2 (±0.95)	43.2 (±0.8)	2.0 (±0.15)	4.4 (±0.3)
J	4.68	Yes	42 (±1)	50	0.4 (±0.02)	80 (±20)	1.1 × 1011	46.1 (±1.0)	44.1 (±0.7)	2.0 (±0.3)	4.3 (±0.6)

*Ambient*											
K	0.94	–	48 (±1)	57	0.25 (±0.02)	190 (±20)	8.0 × 1011	48.2 (±0.4)	42.1 (±0.3)	6.1 (±0.1)	12.7 (±0.2)
L	1.56	–	47 (±1)	62	0.24 (±0.02)	185 (±20)	9.0 × 1011	49.0 (±0.8)	43 (±0.6)	6 (±0.2)	12.2 (±0.4)

M	0.94	Yes	25 (±1)	110	0.84 (±0.02)	150 (±20)	1.1 × 1012	50.8 (±0.4)	45.6 (± 0.3)	5.2 (± 0.1)	10.2 (±0.3)
N	1.56	Yes	27 (±1)	108	0.78 (±0.02)	145 (±20)	9.9 × 1011	50.3 (±0.3)	44.0 (±0.1)	6.4 (±0.2)	12.7 (±0.1)

a PNDPE layer A is without UV-treatment, B-J UV-illuminated under inert conditions and K-N UV-illuminated under ambient conditions. Samples G-J and M-N are additionally developed.

b *E* is the exposure dose.

c *D* is the thickness of the gate dielectric layer.

d *C_i_* is the capacitance of the gate dielectric layer.

e RMS…root mean square.

f *S* is the subthreshold slope.

g *N_ss,max_* is the calculated values of the maximum interfacial trap density.

h *γ* is the surface energy.

i *γ^P^* and *γ^D^* are the polar and dispersive components of the surface energy.

j Surface polarity is the ratio of the polar component to the surface energy in percent.

The top electrode for the capacitor structures were realized by e-beam evaporation of 50 nm Al through a shadow mask and had an overlap area of about 0.1 cm^2^. The frequency dependence of the gate dielectric capacitance was measured by impedance spectroscopy techniques with an LCR meter (Hioki 3532-50 LCR) and showed consistent results over a frequency range from 42 Hz up to 10 kHz. For the data processing of the oTFT characteristics the capacitance at 1 kHz was used.

Atomic Force Microscopy (VEECO Dimension 3100 AFM) topography images were used to determine the surface roughness of the PNDPE layer and the morphology of the semiconductor. AFM images were analyzed using the free WSxM software from Nanotec Electronica [20]. The pentacene growth on PNDPE was studied by evaporation of pentacene layers onto the preheated samples at 65 °C in high vacuum (Mini-Coater from Tectra with a home built sample heater). The rate for the submonolayer studies was 0.2 nm min^−1^, for the thicker films (50 nm) the rate was increased to 0.6 nm min^−1^ after deposition of 5 nm.

### 2.3 Device fabrication

Organic thin film transistors were fabricated in a staggered bottom-gate architecture according to the layer setup illustrated in Fig. 1a. The gate electrode was processed under high vacuum conditions on pre-cleaned glass slides by e-beam evaporation of a 30 nm thick aluminum layer through a shadow mask at a rate of 1 Å s^−1^. The PNDPE dielectric layer on the aluminum gate electrode was processed in the same manner as mentioned before for the test samples. To demonstrate the ability of the dielectric patterning, the layers were UV-illuminated through a simple metal shadow mask in order to open the gate contact pad as displayed in the inset of Fig. 1a. After UV-illumination the non-exposed areas were easily removed by rinsing the sample with anisole for a few seconds. This process-step is called “development” in the paper.

After spin-coating and patterning (illumination and development) of the dielectric, a 50 nm thick pentacene layer was evaporated onto the preheated samples at 65 °C in high vacuum at a rate of 0.2 nm min^−1^ for the first 5 nm and 0.6 nm min^−1^ for the remaining 45 nm. Source and drain electrodes were deposited by e-beam evaporation of gold through a shadow mask in order to form 50 nm thick contacts. After production, all oTFT samples were protected from light and stored under argon atmosphere. The channel length of the fabricated oTFTs ranged from 70 μm to 240 μm. The channel width ranged from 1.5 mm to 4.4 mm.

### 2.4 X-ray photoelectron spectroscopy (XPS) measurements

XPS measurements were performed with an ultra high vacuum (UHV) surface analysis system from Omicron Nanotechnology, using a monochromated Al Kα_1_ radiation (*hν *= 1486.7 eV) with a take-off angle of 90°.

### 2.5 Electrical characterization

Electrical measurements of the oTFTs were carried out under exclusion of light, using a parameter analyzer from Miedl-Bauer-Technologies. The sampling rate during the recording of the characteristics was 1.9 points per second which is sufficiently low to determine hysteresis effects originated by interface traps.

## 3 Results and discussion

### 3.1 Cross-linking behavior of PNDPE

For the investigation of the photo-induced cross-linking, PNDPE with a number averaged molecular weight (Mn) of 102,300 g mol^−1^ was synthesized by means of the ring-opening metathesis polymerization reaction. This polymer showed a low polydispersity (PDI) of 1.07 and also possessed excellent film forming properties when spin-cast from anisole solutions.

Upon irradiation with UV light, photolysis leads to the cleavage of the aromatic ester in the polymer side chain, resulting in the formation of polymeric acyl radicals and phenoxy radicals (Fig. 1b). Recombination of these two radicals leads to the corresponding photo-Fries product, i.e. ortho-hydroxyketone, whereas the radical coupling reaction of two polymeric acyl radicals results in crosslinking [19]. Consequently, the polymer becomes insoluble in organic solvents upon UV-irradiation.

**Fig. 2:**
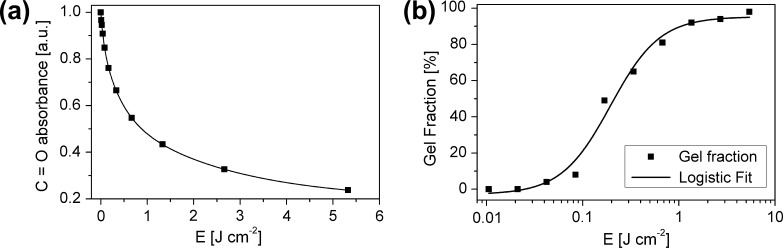
(a) Reaction kinetics of the photo-Fries reaction in PNDPE. (b) Progression of the gel fraction of thin PNDPE films during UV-illumination.

The photoinduced conversion of the aromatic ester group was investigated by Fourier transform infrared spectroscopy (FT-IR). Fig. 2a shows the decrease of the C=O stretching vibration of ester groups at 1745 cm^−1^, observed during the illumination of thin PNDPE films with UV-light of a wavelength of 254 nm. The decrease of the ester groups correlates with the formation of the corresponding acyl- and phenoxy radicals. In a previous study, the degree of recombination, which leads to the formation of the described hydroxyketones (see Fig. 1b), was determined to be 20–25% referred to a quantitative conversion of the ester groups [17]. The residual radicals undergo side reactions such as a decarboxylation reaction or cross-linking of the polymer chains [18], [19].

In order to investigate this cross-linking reaction and its influence on the solubility, a sol–gel analysis was performed. For that purpose, thin films of PNDPE were illuminated for different periods of time and after subsequent development with anisole, the insoluble fraction (gel fraction) was determined using FT-IR spectroscopy by comparing the heights of the ester peak at 1745 cm^−1^ prior to- and after development.

After exposure with an energy density of 1.35 J cm^−2^ (see Fig. 2b) an insoluble fraction of approximately 92% could be observed. Prolonged illumination results in a further increase of the gel fraction towards 100%.

All Fourier transform infrared spectroscopy measurements were done under inert conditions, whereby the subsequent photopattering processes were done under ambient conditions.

It has to be noted that the photo-induced conversion of the aromatic ester groups to the corresponding polar hydroxyketones in the polymer side chain could also cause a change in solubility. In order to exclude this behavior, a further sol–gel analysis was performed using dimethylformamide (DMF) as a solvent. In general, DMF is known as an excellent solvent for polar compounds and polymeric materials. In this experiment a similar cross-linking behavior leading to comparable values of the insoluble fraction was observed. This fact excludes solubility changes due to the formation of polar products in the polymer side chain.

To demonstrate the versatility of this crosslinking reaction for the structuring of thin PNDPE layers, such films were photopatterned using a mask aligner system equipped with a suitable quartz chromium mask. A subsequent development in anisole leads to the dissolution of the non-crosslinked areas. This approach provides good pattern reproducibility with resolutions in the μm-range (see Fig. 1c). Moreover, the material can be patterned directly with e-beam lithography with resolution in the sub-μm regime (see Fig. 1d).

This direct patterning method offers the advantage that no additional photoinitiator is required and cross-linking can be carried out in a short-term process under ambient conditions with non-halogenated solvents. For a good reproducibility of the results a protection from UV-ozone turned out to be a necessary when using a free standing UV-lamp as was the case during the transistor fabrication processes. Although the results under ambient conditions are very promising, we primarily used inert conditions (purging in Argon) to investigate the UV-induced chemical reaction on the oTFT device performance. The conditions during hard contact photolithography in an ozone protected (inert gas purged) mask aligner are, much more comparable to the results under inert gas than to those under ambient conditions.

### 3.2 Dielectric parameters, surface properties and morphology of PNDPE layers

In order to investigate the influence of the photo-Fries rearrangement, the crosslinking of the polymer chains and the development step on the device performance of oTFTs, large area test samples as well as capacitor structures were fabricated. On these samples different film treatments like UV-irradiation with different energies, with or without development step in anisole were explored, a reference sample with non-irradiated PNDPE layers was also included.

**Fig. 3:**
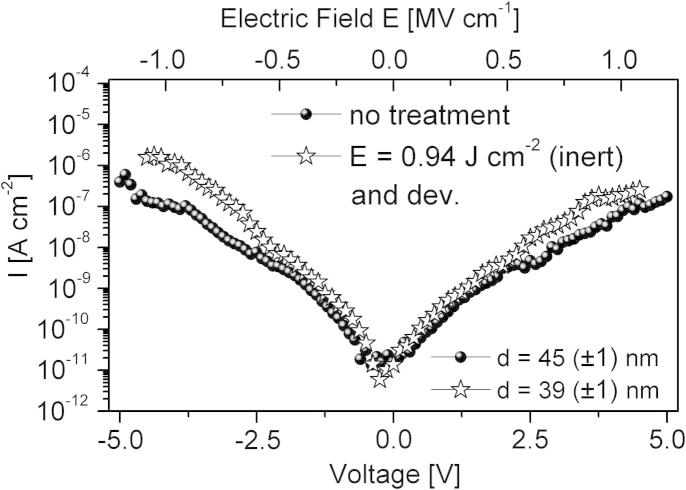
Current leakage through a 45 nm thick untreated and 39 nm thick UV-illuminated (inert, *E *= 0.94 J cm^−2^) and developed PNDPE gate dielectric film in a capacitor structure with an overlap area of 0.1 cm^2^.

The influence of the UV-induced photochemical reaction under inert conditions and the development step on the dielectric parameters was investigated by current–voltage measurements of capacitor structures as displayed in Fig. 3.

Only a very small increase of the leakage current density due to the UV-illumination and the development step is observed, thus showing that the photopatterned PNDPE layers are highly suitable for gate dielectrics in oTFTs. The dielectric parameters of untreated, irradiated and developed PNDPE layers are compiled in Table 1. The layer thickness *d*, verified by a surface profiler, is in the range of 42 nm and shows no significant change upon UV-illumination (inert). For undeveloped samples the DC capacitance *C_i_* slightly increases with the irradiation dose (increasing illumination time from sample B → F in Table 1) which is attributed to an increase in the dielectric constant, being a result of the photo reaction [14]. Additionally, up to a dose of 1.56 J cm^−2^ a decrease of the layer thickness after development is observed leading to an increase of the capacitance. This can be attributed to the incomplete cross-linking of the polymer. For an irradiation dose of 0.39 J cm^−2^ the gel fraction is about 78%. Development of the incompletely crosslinked polymer removes the sol fraction and reduces the polymer thickness by 9 nm (from 42 nm to 33 nm) which is a decrease of about 21% from the original thickness. By irradiation with a dose of *E *= 0.94 J cm^−2^ a cross-linking degree of about 86% is reached (Fig. 2b). After development we measure a 17% reduction of the thickness from 42 nm to 35 nm. These results are in good accordance with the sol–gel analysis of the polymer after UV-illumination displayed in Fig. 2b. Finally, for 1.54 J cm^−2^ exposure dose the gel-fraction is about 95% which is, again, nicely corresponding to the measured reduction of the polymer thickness from 42 nm to 39 nm (7% reduction). Higher doses (4.68 J cm^−2^) induce almost complete cross-linking of the PNDPE dielectric and therefore no decrease of the layer thickness after development is observed. As demonstrated in Table 2 the crosslinking under ambient conditions induces a different behavior due to the not ozone protected PNDPE layers during UV-illumination.

**Table 2:** Extracted transistor parameters for differently treated PNDPE gate dielectric layers.

PDNPE layer^a^	*E* (J cm^−2^)	Dev.	*V_On_* (V)^b^	*V_Thr_* (V)^c^	*S* (mV dec^−1^)^d^	*μ_lin,max_* (cm^2^ V^−1^ s^−1^)^e^	*μ_lin_* (cm^2^ V^−1^ s^−1^)^f^	*I_Off_* (nA)^g^	*W* (mm)/*L* (μm)^h^
*Inert*									
A	–	–	−1.55	−2.4	110 (±20)	0.26	0.26	≈0.1	1.5/70
B	0.39	–	−0.7	−1.6	140 (±20)	0.33	–	≈0.1	1.5/70
C	0.94	–	−0.15	−1.0	150 (±20)	0.31	0.23	≈0.1	1.5/70
D	1.56	–	0.05	−1.05	220 (±20)	0.25	0.17	≈0.1	2/100
E	2.34	–	0.1	−1.0	195 (±20)	0.24	0.15	≈0.1	3.8/200

F	4.68	–	0.1	−0.75	140 (±20)	0.35	0.28	≈0.1	1.5/70
G	0.39	Yes	−1.3	−1.8	100 (±20)	0.15	0.13	≈0.1	4.4/220
H	0.94	Yes	−0.2	−1.15	100 (±20)	0.26	0.22	≈0.1	1.5/70
I	1.56	Yes	−0.1	−0.9	110 (±20)	0.31	0.26	≈0.1	2/100
J	4.68	Yes	−0.2	−1.0	80 (±20)	0.29	0.24	≈0.1	3.2/175

*Ambient*									
K	0.94	–	0.2	−0.65	190 (±20)	0.61	0.49	≈0.1	3.2/175
L⁎	1.56	–	0.45	−0.4	185 (±20)	0.82	0.57	≈0.1	3.2/175

M	0.94	Yes	−0.4	−0.85	150 (±20)	0.14	0.12	≈0.1	3.2/175
N⁎	1.56	Yes	−0.05	−0.8	145 (±20)	0.17	0.15	≈0.1	3.2/175

a PNDPE layer A is without UV-treatment, B-J UV-illuminated under inert conditions and K-N UV-illuminated under ambient conditions. Samples G-J and M-N are additionally developed.

b *V_On_* is the onset voltage.

c *V_Thr_* is the threshold voltage.

d *S* is the subthreshold swing.

e Linear mobility @ *V_GS_* = −4.5 V and @ *V_GS_* = −3.5 V for sample B.

f Linear mobility @ *V_GS_* − *V_On_* = −2.95 V.

g Drain leakage current when the transistor is off. This current is called off-current.

h *W* is the channel width and *L* is the channel length of the oTFT.

⁎ Devices with hysteresis. *V_On_* and *V_Thr_* are extracted from the forward curve.

The surface energy of the photopatternable polymer was determined via the Owens–Wendt–Rabel–Kaelble method, using contact angles of different liquids with known disperse and polar fractions of the surface tension [21]. The extracted surface energy values from the treated and untreated PNDPE layers and their dependence on the irradiation dose are summarized in Table 1. Due to UV-illumination a small change of the surface polarity is observed. The slight UV-induced increase in polarity from 4.3 (±0.4)% to a maximum of 6.0 (±0.4)% can be attributed to the photo-Fries rearrangement, resulting in the generation of hydroxyl groups. Another important observation is that after the development step the polarity decreases to ≈4.4%, independent on the UV-exposure dose which has been applied to the PNDPE-layers.

For comparison, also UV-illuminated PNDPE layers prepared under ambient conditions and therefore presumably surface activated by ozone generated in the UV-light, were investigated by contact angle measurements. Not surprisingly, the polarity of these UV-illuminated PNDPE layers turned out to be in the range of 12.5%, resulting in a higher total surface energy. Interestingly, no significant change of the polarity is observed after development.

**Fig. 4:**
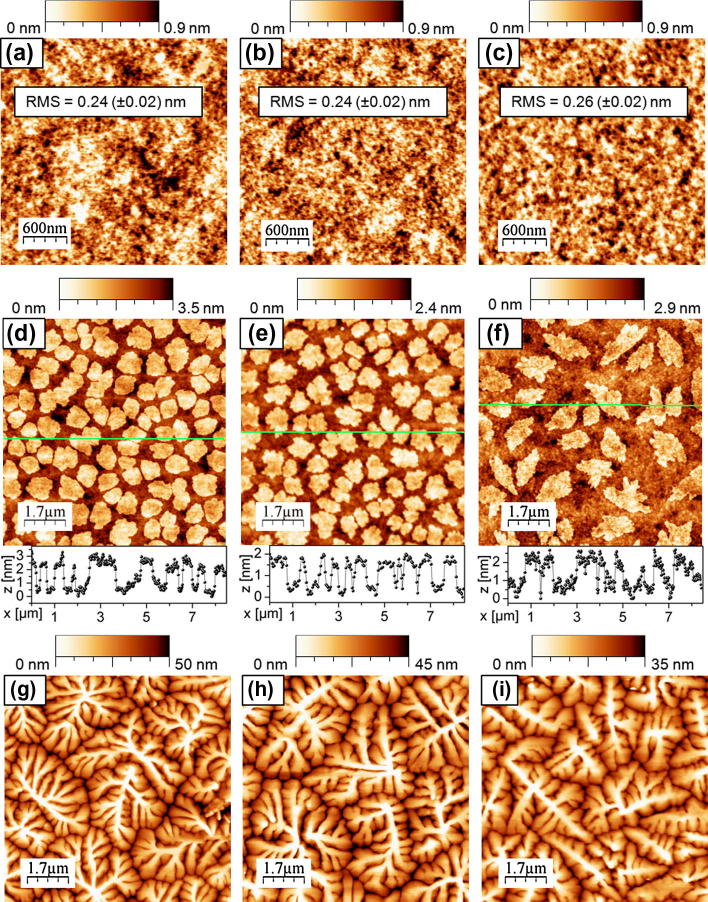
AFM images of (a) untreated PNDPE and (b) after UV-illumination (inert conditions) with an exposure dose of *E *= 0.94 J cm^−2^ and (c) after UV-illumination (inert, *E *= 0.94 J cm^−2^) and development. AFM images of submonolayer pentacene films on (d) untreated PNDPE (e) after UV-exposure (inert) with an exposure dose of 0.94 J cm^-2^ and (f) after exposure (inert, *E *= 0.94 J cm^−2^) and development. 35–50 nm thick pentacene deposited on (g) untreated PNDPE (h) after UV-illumination (inert, *E *= 0.94 J cm^−2^) and (i) after UV-illumination (inert) and development.

**Fig. 5:**
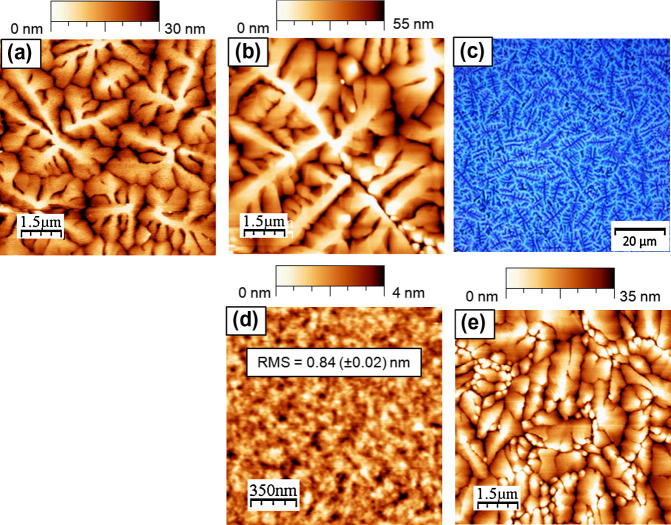
(a) AFM image of pentacene growth on PNDPE after UV-illumination (inert, *E *= 2.34 J cm^−2^). (b) AFM image and (c) micrograph image of pentacene growth on PNDPE after UV-illumination under ambient conditions with an exposure dose of 1.56 J cm^−2^. (d) AFM image of PNDPE after UV-illumination (ambient conditions, *E *= 0.94 J cm^−2^) and development and (e) pentacene growth on PNDPE after UV-illumination (ambient, *E *= 0.94 J cm^−2^) and development.

The influence of the PNDPE treatment on the surface roughness of the dielectric layer was analyzed by means of the AFM height images displayed in Figs. 4a–c, 5d and listed in Table 1. The UV-irradiation step and the development of the PNDPE layers under inert conditions leave the roughness unchanged as indicated by the Root Mean Square (RMS)-roughness values that remain within the error tolerance. The average surface roughness of the untreated and the UV-exposed polymeric dielectrics illuminated under inert and ambient conditions is between 0.24 (±0.02) nm and 0.28 (±0.02) nm. After development a slight increase with irradiation dose up to 0.4 (±0.02) nm is observed for samples UV-exposed under inert conditions and a RMS in the range of 0.8 (±0.02) nm is measured for developed samples UV-exposed under ambient conditions. This increase can be attributed to a removal of photooxidation side products by the solvent. More side products are expected for non-ozone protected UV-illumination.

### 3.3 Pentacene film growth on differently treated PNDPE

Concerning the morphology of the pentacene layer formed on these dielectric layers (UV-expose under inert conditions), a high degree of similarity is observed for untreated, illuminated, and developed PNDPE layers with maximum grain sizes typically reaching up to 4.5 μm. The large grain size and the similarity in morphology can be attributed to the very smooth surfaces of both, on untreated (Fig. 4g) and on treated (Fig. 4h and i) PNDPE layers, which is in agreement with several reports from literature [22], [23], [24], [25]. Consistently, the grain size is also independent of the irradiation dose (Fig. 5a). It is commonly believed that the substrate surface energy is a crucial factor for the crystallographic growth of small molecule based thin films [25], [26], [27]. According to the data presented by Yang et al. [26] the ratio of the overall surface energy of the substrate to that of the pentacene molecules determines the growth mode. For high energy surfaces the 2D growth mode (Stranski–Krastanov) is expected, since the approaching pentacene molecules are more tightly bound to the substrate than to each other and therefore condense into a monolayer. Referring to a total surface energy of approximately 45 mJ m^−2^, with virtually no polar component [26], it is clear that all PNDPE layers can be treated as high energy surfaces, thus supporting 2D growth. This is consistent with our observation of similar morphology and grain size for less polar PNDPE surfaces (<6%). It has to be mentioned that on sample K and L with a 12% polarity, stemming from UV-ozone due to illumination in air, very large pentacene grains with dimensions up to 10 μm were observed (Fig. 5b and c). This can be attributed to the substantial polarity of this surface resulting in a decreased sticking coefficient that further shows up as a strong decrease of the nucleation density [28], [29]. The promising semiconductor morphology on PNDPE layers which were UV-crosslinked in air actually resulted in devices with high charge carrier mobility. However, the increased dielectric surface roughness over 0.8 nm (Fig. 5d) after development induced smaller crystallite size (≈250 nm–1.5 μm) (see Fig. 5e) and therefore a reduction in mobility [22].

The appearance of the Stranski–Krastanov growth mode on high-energy PNDPE surfaces is further approved by AFM height images of sub-monolayer films of pentacene (70% coverage, ca. 1.2 nm) on UV-treated (inert), UV-treated (inert) and developed and untreated PNDPE dielectrics (see Fig. 4d–f). The height profiles indicate the formation of two-dimensional islands with a step height of about 1.4–2 nm close to the interplanar spacing of pentacene unit cell in the thin-film phase (15.5 Å). No evidence to 3D growth is found, although the formation of the second monolayer has already started before completion of the first monolayer coverage – an observation that is typical for low-rate pentacene growth on common dielectric substrates with low to medium nucleation densities [30].

### 3.4 Electrical testing of pentacene based oTFTs with PNDPE dielectric layer

**Fig. 6:**
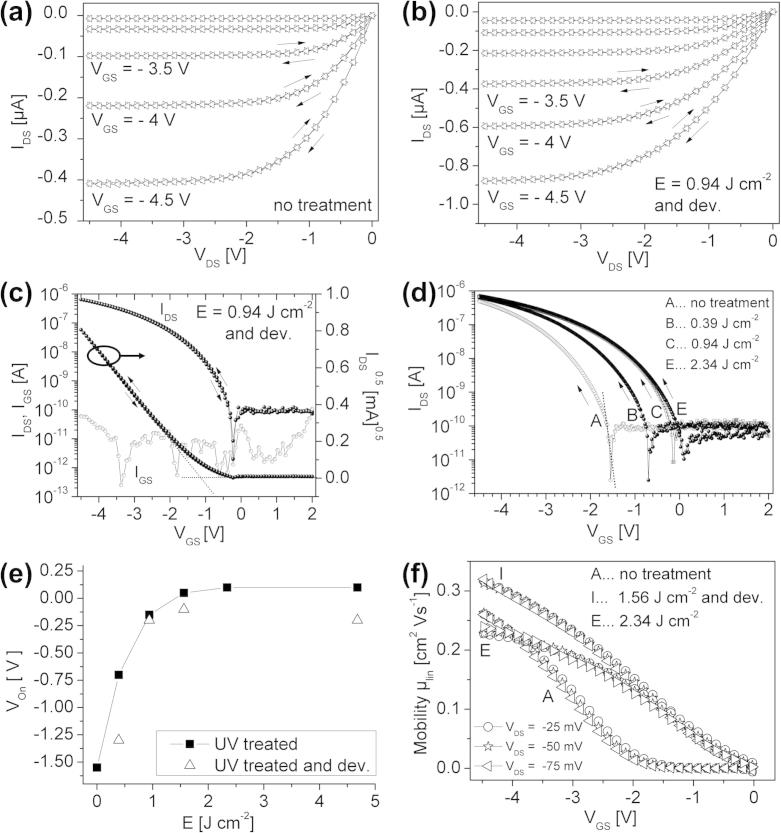
Electrical characteristics of pentacene oTFTs with channel length *L *= 70 μm and a channel width of *W *= 1.5 mm. Output characteristic with an (a) untreated PNDPE-layer as a gate dielectric and (b) after UV-illumination (inert, 0.94 J cm^−2^) and development. (c) Transfer characteristic *I_DS_* versus *V_GS_* at a drain voltage of −1.5 V of a UV-illuminated (inert, *E *= 0.94 J cm^−2^) and developed PNDPE-layer as a dielectric in a pentacene oTFT recorded for forward and reverse direction of the gate voltage sweep. (d) Transfercharacteristics for different UV-exposure dose of PNDPE gate dielectric. (e) Dependence of the onset voltage *V_On_* on the irradiation dose *E*. (f) Gate voltage variation of the charge carrier mobility extracted from the linear regime (*V_GS_* ≪ *V_DS_*) for untreated, UV-illuminated (inert, *E *= 2.34 J cm^−2^) and UV-illuminated (inert, *E *= 1.56 J cm^−2^) and developed PNDPE-layers at three different drain voltages (*V_DS_ *= −25 mV, −50 mV, −75 mV).

The electrical characteristics of oTFTs samples without UV-illuminated, with UV-illuminated (inert) and illuminated (inert) and developed gate dielectric layers are plotted in Fig. 6. The critical device parameters are extracted from these curves and listed in Table 2. The output characteristics (Fig. 6a and b) show a clear saturation of the drain current and a quadratic increase of the saturation drain current level with the gate bias for the devices with non-illuminated (sample A) and illuminated and developed (sample H) dielectric layers. Neither for the UV-treated nor for the untreated samples any drain current hysteresis between forward and reverse drain voltage sweep is observed. The drain current level of sample H (Fig. 6b) is significantly higher, which can be assigned to a positive shift of the onset/ threshold voltage.

In Fig. 6c both, the *I_DS_*(*V_GS_*) and *I_GS_*(*V_GS_*) of sample H, are plotted in semi-logarithmic representation as well as the square root of the drain current as a function of the gate bias. From the former an onset voltage *V_On_* = −0.2 V, a subthreshold swing *S *= 0.1 V dec^−1^, a threshold voltage and a gate leakage current of around 10 pA can be extracted, whereas from the latter curve the threshold voltage *V_Thr_* = −1.15 V (dashed line) is derived. Again,no hysteresis is observed. The subthreshold swing S is the inverse of the subthreshold slope extracted as the maximum slope of the (quasi)linear part of the subthreshold current (dashed line in the semilogarithmic plot of the transfer curve, as shown Fig. 6d).

The transfer characteristics of transistors with differently UV-treated gate dielectrics are compared in Fig. 6d and reveal *S *= 0.11 V dec^−1^ and *V_On_* = −1.55 V for sample A, *S *= 0.14 V dec^−1^ and *V_On_* = −0.7 V for sample B, *S *= 0.15 V dec^−1^ and **V_On_* *= −0.15 V for sample C, and *S *= 0.195 V dec^−1^ and *V_On_ *= 0.1 V for sample E. Obviously, the UV-illumination induces a continuous shift of the onset voltage towards more positive values with increasing dose (Fig. 6e). The shift levels off for high dose values, but does never reach the value of the untreated sample (A). The same observations hold for the threshold voltage.

**Fig. 7:**
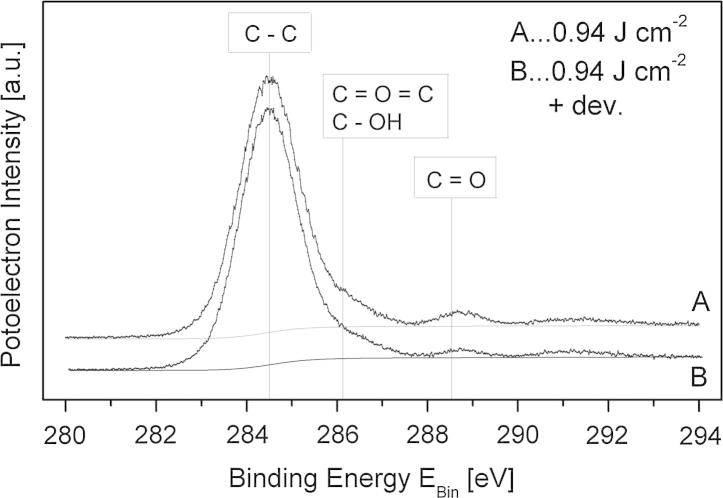
C 1s XPS spectra for (A) UV-illuminated (inert conditions) and (B) UV-illuminated (inert conditions) and developed PNDPE layers. The positions of the chemical bonds are indicated in the plot.

The UV-illumination also influences the subthreshold swing, but there is no continuous increase with irradiation dose and after development the swing decreases to values in the range of the untreated sample. The decrease of the swing after development can be explained by removing recombination products from the photoreaction with carbon–oxygen bonds from the dielectric surface by the solvent which leads to a reduction of interface traps. The reduction of C—O double bonds (Ketone, Ester) at the surface after development was clearly verified by additionally performed X-ray photoelectron spectroscopy (XPS) studies which provide a plausible explanation for the lower polar fraction of the surface energy and reduction of the subthreshold swing after development (see Fig. 7). Additionally the swing seems to be closely correlated with the surface polarity of the dielectric layer showing a maximum for intermediate irradiation dose values (see Table 1). The correlation between surface polarity and swing holds for devices with untreated, UV-treated (inert) and UV-treated (inert) and developed PNDPE layers.

In all samples, treated or not treated, the off-current level is in the range of 0.1 nA. The dependence of the charge carrier mobility on the gate voltage of the untreated sample (sample A), of a sample after UV-irradiation (sample E) and of a sample after UV-irradiation and development (sample I) is displayed in Fig. 6f. The developed sample I and the untreated sample A show the quasi-linear *μ*(*V_GS_*) dependence that is often observed in oTFTs with polymer gate dielectrics [31], [32], [33]. At *V_GS_* = −4.5 V the untreated oTFT has a linear field effect mobility *μ_lin_* of 0.26 cm^2^ V^−1^ s^−1^ whereas for sample E the field effect mobility *μ_lin_* is 0.24 cm^2^ V^−1^ s^−1^ and for sample I *μ_lin_* is about 0.31 cm^2^ V^−1^ s^−1^. No dependence of the mobility on the drain voltage and thus the lateral electric field is observed (see Fig. 6f). In order to find out whether the difference in mobility between oTFTs with illuminated (inert), illuminated (inert) and developed and non-illuminated dielectrics is more related to a difference in the onset voltage than to a difference in the effective mobility (affected by trapping, scattering from defects, grain boundary related potentialbarriers or contact resistance) the transfer characteristics was plotted with respect to (*V_GS_* − ). At *V_GS_* − **V_On_* *= −2.95 V the as-determined effective mobility values for illuminated (inert) and developed samples are quite comparable (see Table 2), whereas UV-illumination alone seems to induce a decrease of the effective mobility except for the sample with the highest dose (sample F). This decrease originates from the decreased slope of *μ*(*V_GS_*) at **V*_GS_ *> −3 V (see sample E in Fig. 6f) that typically arises from a super-linear current increase in the *I*(*V*) curves at *V_GS_* ≫ *V_DS_* and is often attributed to an increased contact resistance [33].

From the measured subthreshold swing the upper limits of density of interfacial trap states *N_ss,max_* can be extracted according to the method reported by Rolland et al. [34]. For a rough assumption that the density of deep bulk states *N_bs_* and interface states *N_ss_* is independent of energy, the subthreshold slope *S* is linked to *N_bs_* and *N_ss_* by

(1) $$S=\frac{\mathrm{kT}}{q\log(e)}\left[1+\frac{{\mathrm{qd}}_{i}}{\varepsilon_{i}}\left(\sqrt{\varepsilon_{s}N_{\mathrm{bs}}}+{\mathrm{qN}}_{\mathrm{ss}}\right)\right]$$

where *ɛ_i_* and *ɛ_s_* are the insulator and semiconductor dielectric constants, respectively, *d_i_* the dielectric thickness, *k* the Boltzmann constant, *q* the elementary charge and *T* the temperature. Eq. (1) can be used to calculate the upper limit of interface trap density *N_ss,ma_*_x_ by assuming *N_bs_ *= 0 which leads to

(2) $$N_{\mathrm{ss}\text{,}\max}=\left[\frac{q\log(e)s}{\mathrm{kT}}-1\right]\frac{C_{i}}{q^{2}}$$

where *C_i_* is the polymeric gate dielectric capacitance.

Based on the measured swing values the calculated interface trap densities from Eq. (2) are compiled in Table 1. A upper density limit of *N_ss,max_ *= 2.7 × 10^11^ cm^−2^ eV^−1^ for the untreated polymer (sample A), a higher upper limit in the range of 4.3 × 10^11^ cm^−2^ eV^−1^ to 8.6 × 10^11^ cm^−2^ eV^−1^ for UV-illuminated samples (B–F) and a upper limit in the range of 1.1 × 10^11^ cm^−2^ eV^−1^ to 3.0 × 10^11^ cm^−2^ eV^−1^ for UV-illuminated and developed polymer layers (G–J) is extracted. For a typical trap energy range of 0.2–0.6 eV the number of interface trap states is in the order of only 10^10^ cm^−2^. The extracted *N_SS_* values for PNDPE based oTFTs are low, in fact they are much smaller than the average interface trap density of states observed in amorphous silicon TFTs being in the range of 10^12^ cm^−2^ eV^−1^ [34]. The upper trap density limit for the samples prepared under ambient conditions (K–N) is in the range of 8.0 × 10^11^ cm^−2^ eV^−1^ to 1.1 × 10^12^ cm^−2^ eV^−1^ and therefore much higher compared to the inert fabricated samples, this difference again is attributed to photooxidation side products created by the ozone.

The sampling rate during the recording of the characteristics was 1.9 points per second which is sufficiently low to determine hysteresis effects originated by interface traps. In fact with this quite slow sampling rate no hysteresis effects are observed for both, the treated and the untreated samples which confirm the low calculated trap densities.

Based on all these observations it is clear that the dominant influence of the UV-illumination on the device characteristics is the shift of the onset/threshold voltage towards positive values occurring for transistors with developed and non-developed dielectrics (see Fig. 6e). The *V_Thr_*-shift does not correlate with the interface trap density and therefore cannot be attributed to hydroxyl group related electron traps at the surface. The change in the onset/threshold voltage therefore is attributed to negative charges originated from the hydroxyketone groups or phenols in the polymer which are products of the photoreaction during UV-illumination. These negative charges are either fixed or deep trapped electron charges that can’t be recharged by the gate bias sweep and therefore do not contribute to the swing [35]. The number of these negative charges can be estimated via

(3) $$\Delta N_{\mathrm{if}}=\frac{\Delta V_{\mathrm{Thr}}\cdot C_{i}}{q}$$

amounting to an upper limit for the number of generated charges Δ*N_if_ *= 6.5 × 10^11^ cm^−2^ at the maximum observed voltage shift of Δ*V_Thr_*= + 1.65 V.

The overall interface trap density in the PNDPE-based transistors is low, irrespective of the treatment and therefore no hysteresis is observed. The sampling rate during the recording of the characteristics was 1.9 points per second which is sufficiently low to determine hysteresis effects originated by interface traps. The increase of the interface trap density of states observed for the UV-illuminated under inert conditions and not developed samples (B–F) can be attributed to residuals of recombination products from the photoreaction on the dielectric surface (see above) and disappear for the developed samples (G–J). The decrease in mobility observed for some devices is not directly correlated to grain boundary effects since the morphology of pentacene on illuminated and non-illuminated PNDPE layers is identical but presumably is resulting from an increased contact resistance in not fully cross-linked samples.

**Fig. 8:**
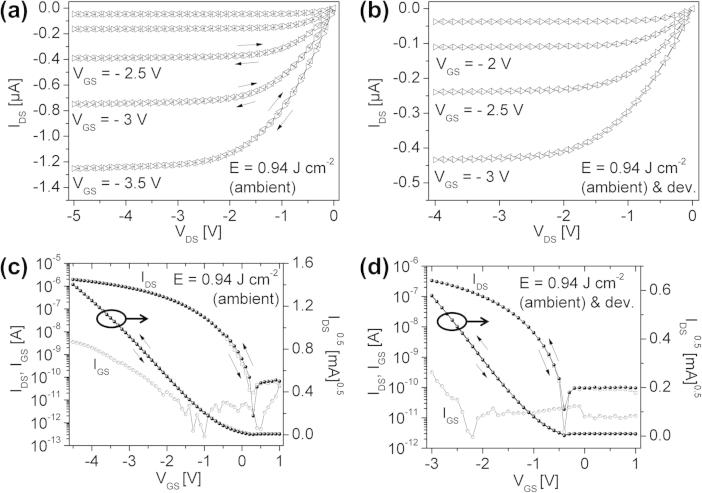
(a) Output characteristic and (c) transfer characteristic of a UV-illuminated (ambient conditions, *E *= 0.94 J cm^−2^) PNDPE-layer as a dielectric in a pentacene oTFT. (b) Output characteristic and (d) transfer characteristic of a UV-illuminated (ambient conditions, *E *= 0.94 J cm^−2^) and developed PNDPE-layer as a dielectric in a pentacene oTFT.

As a concluding remark, the oTFTs with dielectric layers illuminated under ambient conditions also showed sufficient device performance with high charge carrier mobility (up to 0.8 cm^2^ V^−1^ s^−1^). The electrical characteristics of these oTFTs are plotted in Fig. 8 (samples K and M). However, a strong correlation between the exposure time (generation of ozone-induced reaction products on the surface) and hysteresis effects could be observed at UV-doses >1 J cm^−2^. As mentioned above the increase of surface roughness after development leads to smaller pentacene crystallites and a reduction in mobility.

## 4 Conclusions

The photo-Fries rearrangement of the polymer poly((±)endo,exo-bicyclo[2.2.1]hept-5-ene-2,3-dicarboxylic acid, diphenylester) (PNDPE) under UV-illumination at 254 nm is accompanied by a selective cross-linking of the macromolecules, leading to a change in solubility in the non-halogenated organic solvent anisole. This has been verified by sol–gel analysis as well as by an investigation of the dielectric layer thickness as a function of illumination dose and development. The decrease of the dielectric layer thickness after development directly correlates with the dose controlled gel fraction. PNDPE layers can be photopatterned with resolutions in the low μm-range without the need for any additional photoinitiator. A protection from UV-ozone influence during the UV-exposure is useful with respect to reproducibility. This condition can be achieved by inert gas purged maskaligner in contact mode photolithography.

Low-voltage pentacene based oTFTs were fabricated on ultrathin and electrically dense photopatterned PNDPE layers. The influence of the photopatterning process on the dielectric and the surface properties as well as on the device characteristics was investigated in detail.

Capacitor structures with treated (UV-illuminated, UV-illuminated and developed) and untreated PNDPE layer shows excellent dielectric properties for the usage in oTFTs. A low leakage current of ≈10^−6^ A cm^−2^ at 1 MV cm^−1^ for 39 nm thick treated PNDPE layer, a capacitance of ≈50 nF cm^−2^ and smooth surfaces with RMS in the range of 0.25 nm are shown. On all treated and untreated PNDPE gate dielectrics a Stranski Krastanow growth mode of pentacene was found verified by submonolayer growth investigation by means of AFM measurements.

It turned out that the changes in surface energy and roughness, which are induced by a long-term UV-illumination under inert conditions are negligible and do not influence the semiconductor morphology or the mobility. PNDPE-layers which were UV-illuminated in ambient conditions show a significant change in surface energy and roughness. This leads to a change in semiconductor growth and mobility and can be attributed to the impact of UV-ozone on the unprotected polymer layer in our experimental setup. Nevertheless, hysteresis free oTFTs with a mobility up to 0.8 cm^2^ V^−1^ s^−1^ (but less reproducible) could be shown under these conditions.

However, negative charge carrier traps are generated by the photoinduced rearrangement reaction leading to a stable positive threshold voltage shift for devices with fully cross-linked dielectric layers. The development step is leading to a slight reduction of surface polarity as well as interface trap density by removing polar recombination products from the photoreaction. The overall interface trap density in the PNDPE-based transistors is very low, irrespective of the treatment and therefore no hysteresis is observed. Slight changes in the transistor performance (subthreshold swing, onset and threshold voltage) can be directly correlated to the chemical modification of the polymer upon UV-irradiation. oTFTs with photopatterned fully cross-linked PNDPE layers thus have very stable characteristics with low off-currents, very few interface traps, no hysteresis, a reasonably high charge carrier mobility up to 0.35 cm^2^ V^−1^ s^−1^, low off-currents and a small negative threshold voltage of around −1 V.
